# Comparative Genomic and Transcriptomic Analysis Provides New Insights into the Aflatoxin B_1_ Biodegradability by *Kocuria rosea* from Deep Sea

**DOI:** 10.3390/microorganisms13040875

**Published:** 2025-04-10

**Authors:** Jingying Wang, Jun Nan

**Affiliations:** State Key Laboratory of Urban Water Resource and Environment, School of Environment, Harbin Institute of Technology, Harbin 150090, China

**Keywords:** comparative genomic, transcriptomic, *Kocuria*, *Kocuria rosea*, aflatoxin B_1_

## Abstract

As a mycotoxin, aflatoxin B_1_ (AFB_1_) has attracted increasing attention due to its highly toxic effects, such as teratogen, carcinogen, and mutagen. AFB_1_-producing fungi have been found in both terrestrial and marine sources. Over the last two years, the marine-derived bacteria *Kocuria rosea* has shown the ability to degrade AFB_1_. However, no studies have yet explored the aflatoxin degradation potential in the genus *Kocuria*, and the molecular basis of aflatoxin metabolisms by strain has not yet been investigated. In this study, we first compared and analyzed the genomic features of 13 bacteria of the genus *Kocuria* by comparative genomic analysis and investigated the evolutionary patterns (evolutionary selection pressure analysis and gene family expansion analysis) and pan and core genomes of *Kocuria* strains. It was found that *Kocuria* genus strains showed high potential in the bioremediation of aromatic compounds and aflatoxin degradation. In addition, this study revealed 1421 differentially expressed genes and 752 upregulated genes between the aflatoxin group and the control group through transcriptome analysis and conducted functional annotation and analysis of functional enrichment. According to the expression and functional analysis of upregulated genes, the enzymes and genes (cytochrome P450, oxidoreductase, intradiol ring-cleavage dioxygenase, hydrolase, and monooxygenase) involved in the aflatoxin degradation of *Kocuria rosea* were screened. These findings indicate the potential of *Kocuria rosea* in the remediation of aflatoxin contamination and provide a foundation and direction for the further excavation of novel degradation genes, exploration of degradation mechanisms, and genomic modification.

## 1. Introduction

The oceans, which account for 70% of the Earth’s surface, are one of the most vital ecosystems on the Earth and contain abundant microbial resources [1]. Bacteria and archaea play a crucial part in the biogeochemical cycle of carbon, oxygen, nitrogen, phosphorus, sulfur, and iron in the ocean [2]. Marine microorganisms have a wide range of applications in bioremediation in addition to their ecological role. The gene encoding catechol 2,3-dioxygenases, which is considered a marker for the identification of aromatic hydrocarbon- (AH) and polycyclic aromatic hydrocarbon (PAH)-degrading bacteria, has been detected in marine species of different genera, such as *Pseudomonas* [3], *Neosphingomonas* [4], *Celeribacter* [5], and *Cycloclasticus* [6]. Cao et al. [5] found that *Celeribacter indicus* P73T isolated from deep-sea sediments contained 138 genes related to aromatic compound degradation, including 14 genes encoding dioxygenase genes. In addition, *Celeribacter indicus* P73T can also produce a 7,8-fluoranthene dioxygenase from the upstream fluoranthene degradation pathway. Marine bacteria and marine fungi isolated from the ocean and *Zalerion maritimum* have been shown to degrade environmental pollutants such as polymer films [7] and polyethylene [8]. A novel deoxynivalenol-degrading bacterium, *Pelagibacterium halotolerans* ANSP101, was isolated from seawater in the Bohai Sea, China [1]. Mining new metabolic pathways, secondary metabolites, pollutant-related degradation genes, and degrading enzymes from marine microorganisms have potential application value in enzyme technology, synthetic flora, bioremediation, and other aspects.

AFB_1_ is a derivative of dihydrofuran oxonaphthalen-octanone containing a bifuran ring and an oxonaphthalen-octanone (coumarin). Substances with structural similarity to AFB_1_, such as the PAH naphthalene [9] and coumarin structural analogs, are widely distributed in the marine environment. Many coumarin-based compounds have been identified in the ocean, such as coastal plants, marine bacteria, marine fungi, and invertebrates. For instance, 3-Iminocoumarin and dozens of other metabolites were detected in mangrove fungi from the South China Sea [10]. The 3-aminocoumarins, such as trichodermin A, trichodermin B, and aspergillazine A, were isolated from different marine-derived fungi *Trichoderma virens* and *Spicaria elegans* for the first time in the early 21st century [11,12,13]. Trichodermamide C and hatsumamide A and B were isolated in the fungus *Penicillium steckii* from the deep sea by physical and chemical screening [14]. In addition, four furancoumarin compounds were identified from the fungus *Penicillium* sp. from the South China Sea [15]. Four benzo coumarins and their derivatives have also been found in marine sources [16]. In addition to the above coumarin analogs within the three rings, the pentacyclic coumarin analog aflatoxins have been found in the ocean. Different aflatoxins have been found in the marine fungus *Aspergillus flavus* and in seaweed [17,18]. Additionally, *Emericella venezuelensis*, which was reported to produce aflatoxins, is of marine origin [19]. Therefore, microorganisms that can degrade coumarin analogs and AFB_1_ are likely to exist in the marine environment.

Aflatoxin is a mycotoxin first uncovered after the prevalence of ‘turkey X disease’ in London, UK. In 1960, more than 100,000 turkeys in the UK showed severe enteritis and hepatonecrosis within months, followed by sudden death over a period of days or weeks [20,21]. The cause of the disease was that turkeys had eaten moldy peanut powder from Brazil, and it was found that the metabolite of *Aspergillus flavus*, aflatoxin, was the main cause of the high toxicity. AFB_1_ is the most toxic aflatoxin and is capable of causing genetic mutations, cancers, reproductive health defects, immunosuppression malnutrition, and growth impairment [22,23]. AFB_1_ is the only mycotoxin classified as a Group I carcinogen by the International Agency for Research on Cancer (IARC) in 2002 [24]. Therefore, solving the problem of aflatoxin contamination has gradually attracted widespread attention. In terms of biological detoxification, AFB_1_ can be removed or degraded by adsorption, fungal degradation, bacterial degradation, and enzyme action. According to Rahaie et al. [25], *Lactobacillus rhamnosus* could absorb 90% of the AFB_1_ in pistachios and had no impact on the quality characteristics of pistachios, such as color, texture, and peroxide value. Moreover, it was also revealed that the fungi *Pleurotus eryngii* and *Pleurotus ostreatus* could effectively degrade AFB_1_, with degradation rates of 86% and 91% achieved in 28 and 15 days, respectively, at 30 °C [26,27]. Wang et al. [28] isolated an AFB_1_-degrading strain, *Escherichia coli* CG1061, from chicken cecum, which degraded 93.7% of AFB_1_ within 72 h. Additionally, some enzymes such as laccase [29], manganese peroxidase [30], F_420_H_2_-dependent reductase [31], and oxidase [32] have been identified to have activity in degrading AFB_1_ [33]. Most of the degrading bacteria are isolated from terrestrial sources, derived from cow dung [34], soil [35], fodder [36], crops [37], contaminated maize [38], and tree trunks [27]. However, unknown aflatoxin-degrading bacteria may exist in the marine environment. In the past two years, the bacteria *Kocuria rosea* from the deep sea has been found to degrade AFB_1_, and 10 degradation products have been identified [33].

*Kocuria* are Gram-positive bacteria belonging to the family *Micrococcaceae*, order *Actinomycetes*, and class *Actinobacteria*. The genus *Kocuria* was previously categorized as *Micrococcus* spp., but after phylogenetic analysis, a taxonomic alteration was performed based on 16s r DNA sequences and the amino acid composition of peptidoglycans [39,40]. Bacteria of the genus *Kocuria* have been explored in bioremediation for years. Researchers isolated native calcifying bacteria *Kocuria flava* from soil in the mining area of Urumqi, China, and found its ability to remediate copper-contaminated soil [41]. Additionally, *Kocuria varians* can produce flavin-dependent putrescine oxidase that can degrade putrescine in wine [42]. For *Kocuria rhizophila*, it has been found that endophytic bacteria isolated from the high accumulator Oxalis corniculata can absorb cadmium and chromium in aqueous solution [43]. Furthermore, the application of *Kocuria rhizophila* significantly affected the growth promotion and metal accumulation capacity of *Glycine max L*. in industrial and cadmium-contaminated (100 and 200 mg kg^−1^) soils [44]. In addition, *Kocuria rosea* was reported as a novel feather-degrading bacterium in 2000 [45] and has the potential to be utilized for the production of nutrients by the fermentation of feathers, and the fermentation products can be used as an inexpensive source of protein in animal feed [46]. Another report demonstrated that peroxidase from *Kocuria rosea* can decolorize industrial dyes and is capable of oxidizing differently substituted phenolics, such as guaiacol, syringol, and methoxyhydroquinone, as well as nonphenolic aromatic molecules such as veratryl alcohol [47]. However, the whole genome of the genus *Kocuria* strains has not been compared and analyzed, and the genomic and functional differences for the genus *Kocuria* have not been explored.

Therefore, this study aims to investigate the differences in genomic features, evolutionary patterns (evolutionary selection pressure analysis and gene family expansion analysis), pan-genomes, and core genomes of 13 *Kocuria* bacteria and explore the biological potential of *Kocuria* bacteria for AFB_1_ degradation in bioremediation. In addition, although previous studies have screened that strain *Kocuria rosea* from deep sea can degrade AFB_1_, and a variety of degradation products have been identified, genes related to AFB_1_ metabolism in *Kocuria rosea* have not yet been mined and investigated. Therefore, in this study, we aimed to investigate the functions of differentially expressed genes and upregulated expressed genes by transcriptomics techniques and screen genes related to aflatoxin degradation in *Kocuria rosea*.

## 2. Materials and Methods

### 2.1. Comparative Genomic Analysis

The whole genome sequencing data of 13 *Kocuria* strains were downloaded from the NCBI (https://www.ncbi.nlm.nih.gov, accessed on 10 July 2024) database, and comparative genomic analysis was performed (Table 1). The software PGAP v1.2.1 with basic local alignment search tool (blastclust v2.2.18 and blastp v2.3.0) was applied with the Markov clustering algorithm and Gaussian factor for pan-genome analysis. The software parameters were set as follows: E-value ≤ 10^−5^, Score ≥ 40, Identity ≥ 50%, and Inflation = 1.5. In addition, in the evolutionary analysis, average nucleotide identity (ANI) analysis was conducted by the software pyani (https://github.com/widdowquinn/pyani, accessed on 11 July 2024) and analysis method ANIm; the *Kocuria rosea* strain was used as the reference genome, and the Ka/Ks value was calculated using the MA algorithm in the software KaKs calculator v3.0 for evolutionary selection pressure analysis. The gene family was obtained by BLAST alignment using the software orthmcl v2.3.3, and the gene family expansion and contraction analysis was conducted with the software COUNT v10.04.

### 2.2. Strain and Chemical Preparation

Single colonies of *Kocuria rosea* were picked from the M2 solid plate, inoculated into the M2 liquid medium containing carbon sources (Table S1 and Figure S1), and placed in a constant temperature shaker at 30 °C for 180 rpm for 48 h to reach the mid-logarithmic phase. The seed culture liquid in the middle of the logarithmic phase was centrifuged at 8000 rpm for 10 min to collect the bacteria. Moreover, the bacteria were washed three times with a sterile M2 medium without a carbon source. After resuspension, the bacteria were inoculated (inoculation amount 1%) into a single-factor variable medium. The different variable mediums were the experimental group named TBA (M2 medium with only carbon sources as AFB_1_) and the control group named TBC (M2 medium with multiple carbon sources, including glucose, sucrose, yeast extract, and peptone). After inoculation, the strains were cultured at 180 rpm on a 30 °C thermostatic shaker to the mid-logarithmic phase. The culture was centrifuged at 8000 rpm for 10 min for cell collection. The cells in the TBA and TBC groups were washed three times separately with M2 medium without a carbon source and adjusted to the same OD_600_. The cells were inoculated into mediums (1 L with three replicates) for the TBA and TBC groups and cultured at 180 rpm on a 30 °C thermostatic shaker for the logarithmic growth phase. The culture was centrifuged at 4 °C and 8000 rpm for 10 min for bacteria cell collection (around 1.0).

### 2.3. Total RNA Extraction and Quality Detection

TRIzol reagent (Thermo Fisher Scientific, Bremen, Germany) was used to extract the total RNA of the bacteria, and DNase I (Takara, Beijing, China) was applied for genomic DNA removal. The concentration and purity of the extracted total RNA were detected by the Nanodrop 2000 microspectrophotometer (Thermo Fisher Scientific, Bremen, Germany), and the integrity of the RNA was detected by agarose gel electrophoresis (1% agarose gel, voltage 5 V/cm, 15 min). The samples were processed according to the fragment analyzer system kit (DNF-471 kit, Agilent, Santa Clara, CA, USA), and the RNA quality number (RQN) value was determined by the Agilent 5300 fragment analyzer system (Agilent, Santa Clara, CA, USA). High-quality RNA samples were subjected to prokaryotic chain-specific transcription and sequencing.

### 2.4. RNA Library Construction, Library Enrichment, and Sequencing

RNA library construction was conducted with a TruSeqTM RNA sample preparation kit (Illumina, San Diego, CA, USA). A ribo-Zero Magnetic kit (Illumina, San Diego, CA, USA) was used to remove the rRNA. The mRNA was randomly cleaved into small fragments of about 200 bp. The double-stranded cDNA was synthesized by reverse transcription with random primers and a SuperScript double-stranded cDNA synthesis kit (Invitrogen, Waltham, MA, USA). The synthesized double-stranded cDNA structure is a sticky end, and End Repair Mix is added to fill it into a flat end, and then, a base is added at the 3′ end to connect the Y-shaped sequencing adaptor. Phusion DNA polymerase (New England Biolabs, Ipswich, MA, USA) was used for PCR amplification, and 15 cycles of amplification were performed for library enrichment. Paired-end RNA sequencing was performed using Illumina HiSeq X Ten (San Diego, CA, USA) after quantification with a TBS380 fluorometer (TurnerBioSystems, Sunnyvale, CA, USA).

### 2.5. Bioinformatics Analysis of Transcriptome Sequencing Data

The high-quality reads obtained by quality cutting of the original data are clean data. Using the sequence alignment software RSeQC v2.3.6, the clean reads after quality control were compared to the reference genome, and then, the gene expression in the samples was quantified. Meanwhile, the sequencing quality was evaluated (Table S2). The expression level of the gene was quantitatively analyzed by RSEM software v1.3.3, and the transcripts per million (TPM) value was used for expression level measurement. The Pearson correlation algorithm and average linkage clustering method and Euclidean distance algorithm were applied in the calculation process, and expression quantity analysis was carried out. After obtaining read counts of the genes, DESeq2 software v1.42.0 was applied for analysis of the differential gene expression. The Bonferroni–Holm multiple test correction method was used to correct the *p*-value obtained by statistics. Gene Ontology v2023.07 (GO; http://www.geneontology.org/, accessed on 11 July 2024) functional enrichment analysis of differential genes and upregulated genes was performed using the software Goatools v1.4.4.

## 3. Results and Discussion

### 3.1. Genome Characteristics of Genus Kocuria Strains

The characteristics of the genomes of 13 *Kocuria* strains are summarized in Table 1. The genome size ranged from 2.8 Mb to 4.5 Mb, with an average size of 3.7 Mb. The average GC content of the genomes of the 13 strains of the genus *Kocuria* ranged from 69.0 to 74.0%, with an average GC content of 72.0%. Among them, *Kocuria marina* had the lowest GC content, and *Kocuria flava* had the highest GC content, which indicated that the genomes of different strains within the genus differed greatly from each other.

### 3.2. Phylogenetic Analysis of Genus Kocuria

**Figure 1:**
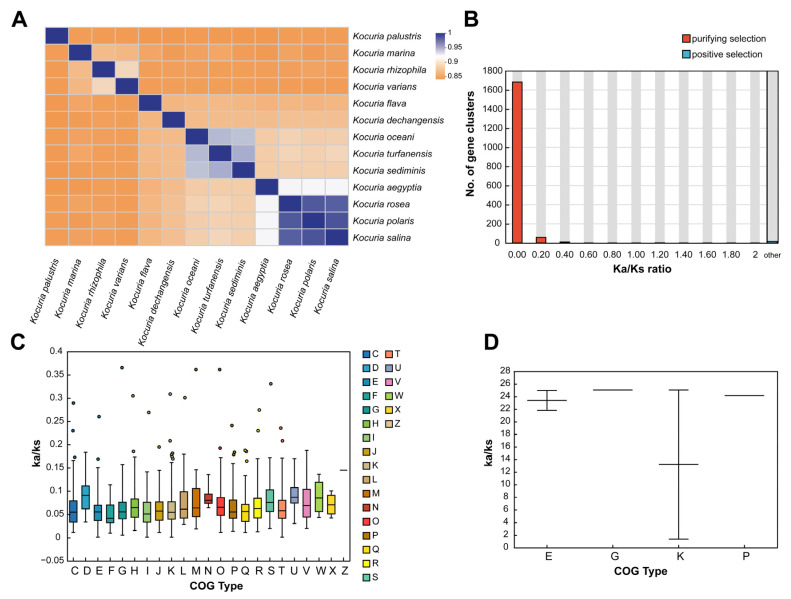
Phylogenetic analysis of the genus *Kocuria*. (**A**) The ANI heat map based on genomes of the genus *Kocuria*. (**B**) Distribution of Ka/Ks. (**C**) COG function selection pressure distribution in the purifying selection. (**D**) COG function selection pressure distribution in the positive selection (C: energy production and conversion; D: cell cycle control, cell division and chromosome partitioning; E: amino acid transport and metabolism; F: nucleotide transport and metabolism; G: carbohydrate transport and metabolism; H: coenzyme transport and metabolism; I: lipid transport and metabolism; J: translation, ribosomal structure, and biogenesis; K: transcription; L: replication, recombination, and repair; M: cell wall/membrane/envelope biogenesis; N: cell motility; O: posttranslational modification, protein turnover, and chaperones; P: inorganic ion transport and metabolism; Q: secondary metabolites biosynthesis, transport, and catabolism; R: general function prediction only; S: function unknown; T: signal transduction mechanisms; U: intracellular trafficking, secretion, and vesicular transport; V: defense mechanisms; W: extracellular structures; X: mobilome: prophages and transposons; and Z: cytoskeleton).

The average nucleotide identity is a method based on digital genome-wide comparison to evaluate the evolutionary distance between bacterial species, which can be used to determine the classification of different *Kocuria* strains. The results of ANI analysis of 13 *Kocuria* genomes are shown in Figure 1A. Among them, *Kocuria rosea* had the highest ANI value in *Kocuria polaris*, *Kocuria salina*, and *Kocuria aegyptia*, which were 98.71%, 98.52%, and 92.48%, respectively, indicating that these four strains had the highest nucleic acid similarity. This is also the reason why their GC content is very close. Therefore, these four strains are more likely to have similar genes and functions.

By analyzing the positive selection of family genes in the evolutionary process, the genes related to the environmental adaptability of the species and their functions can be determined [48,49]. In order to further understand the conservation and evolution of the gene families in the genus *Kocuria*, the evolutionary pressure of each gene family in 13 species of the genus *Kocuria* was measured by calculating the global substitution rate (Ka/Ks) from the non-synonymous substitution rate (Ka) to synonymous substitution rate (Ks) and the number of mutation sites of each gene family under significant negative selection or positive selection. As shown in Figure 1B, 99.04% of the gene clustering Ka/Ks values (Table S3) are less than 1, indicating the *Kocuria* gene family generally has a purification selection effect, which removes harmful mutations to preserve protein structure and function [48] for maintaining the long-term stability of the *Kocuria* genus strains. However, only 17 gene clusters had a Ka/Ks value greater than 1, which indicated a positive selection effect. Among the 17 gene clusters, only five clusters were successfully annotated, which were DNA-binding transcriptional regulators of the PadR family and two permease components of the ABC-type branched-chain amino acid transport system, glycokinases of the NBD/HSP70 family related to carbohydrate transport and metabolism and transcription processes, and ABC-type Fe^3+^ transport system permease proteins related to inorganic ion transport and metabolism.

In addition, the clusters of orthologous groups (COG v2020.11; http://www.ncbi.nlm.nih.gov/COG/, accessed on 11 July 2024) of proteins’ functional selection pressure distribution in Figure 1C showed that the gene family of *Kocuria* had different degrees of purification selection pressure. The genes that experienced the strongest purification selection were genes related to amino acid transport and metabolism (E), lipid transport and metabolism (I), transcription (K), and signal transduction mechanism (T), indicating that these functions are highly conserved, while genes related to defense mechanism (V), cell cycle control, cell division, chromosome partition (D), replication, recombination, and repair (L) are under weak purification pressure. The high Ka/Ks ratio indicated that these genes could obtain specific adaptive mutations related to the acquisition of new functions or adaptive functions. In addition, as shown in Figure 1D, the clustering of gene families containing positive selection effects in *Kocuria rosea* was mainly distributed in amino acid transport and metabolism (E), carbohydrate transport and metabolism (G), transcription (K), and inorganic ion transport and metabolism (P). Among them, there were large differences in the Ka/Ks values among the genes positively selected in term of transcription.

**Figure 2:**
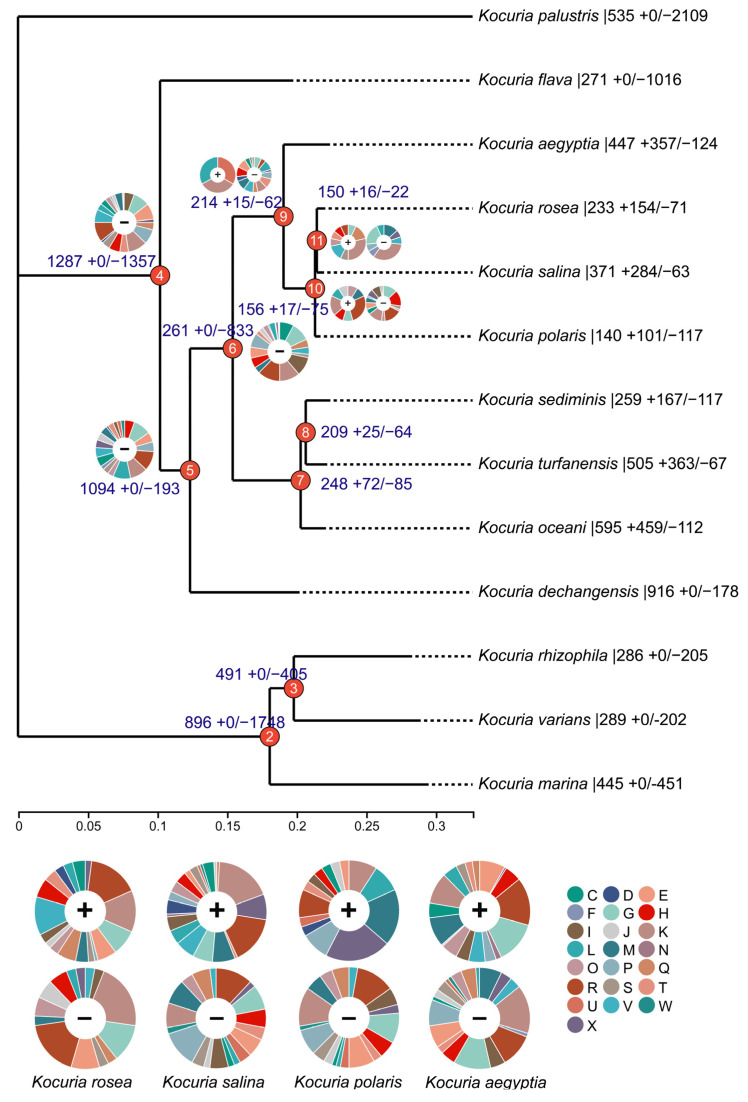
Gene family expansion and contraction evolutionary tree (C: energy production and conversion; D: cell cycle control, cell division and chromosome partitioning; E: amino acid transport and metabolism; F: nucleotide transport and metabolism; G: carbohydrate transport and metabolism; H: coenzyme transport and metabolism; I: lipid transport and metabolism; J: translation, ribosomal structure, and biogenesis; K: transcription; L: replication, recombination, and repair; M: cell wall/membrane/envelope biogenesis; N: cell motility; O: posttranslational modification, protein turnover, and chaperones; P: inorganic ion transport and metabolism; Q: secondary metabolites biosynthesis, transport, and catabolism; R: general function prediction only; S: function unknown; T: signal transduction mechanisms; U: intracellular trafficking, secretion, and vesicular transport; V: defense mechanisms; W: extracellular structures; and X: mobilome: prophages and transposons).

To decipher the evolutionary history of the *Kocuria* species, the identified gene families were mapped onto a core gene tree by COUNT v10.04 software to predict gene family gain, loss, expansion, and contraction (Figure 2). The numbers on each branch of the tree in Figure 2 represent the total number of gene families for each differentiated node and the number of gene families that gain (+) or lose (−) compared to their nearest strains. The pie chart shows the number of genes obtained that are classified by COG category. A large number of gene cluster loss events occurred at nodes 4 and 6, and 51.3% and 76.1% of the gene clusters were lost, respectively. Node 4 mainly lost the gene clusters related to transcription (K), translation, ribosomal structure and biogenesis (J), amino acid transport and metabolism (E), and inorganic ion transport and metabolism (P). Node 5 mainly lost the gene clusters related to carbohydrate transport and metabolism (G); transcription (K); and replication, recombination, and repair (L). Node 6 mainly lost gene clusters related to transcription (K), lipid transport and metabolism (I), carbohydrate transport and metabolism (G), and energy production and conversion (C).

A small number of gene loss events occurred at node 9, node 10, and node 11, and 22.5%, 32.5%, and 14.7% of the genes were lost, respectively. With the expansion of the branches at these three nodes, node 9 mainly lost the gene clusters related to amino acid transport and metabolism (E), coenzyme transport and metabolism (H), signal transduction mechanism (T), transcription (K), defense mechanism (V), and cell wall/membrane/envelope biogenesis (M). Node 10 mainly lost the gene clusters related to coenzyme transport and metabolism (H), carbohydrate transport and metabolism (G), transcription (K), and motor elements (X). Node 11 mainly lost gene clusters related to transcription (K), carbohydrate transport and metabolism (G), and cell wall/membrane/envelope biogenesis (M).

Although no gene acquisition events occurred at nodes 4, 5, and 6, gene acquisition events gradually began to occur at nodes 9 and 7. In the branch of node 9, compared to the other three bacteria (*Kocuria rosea*, *Kocuria salina*, and *Kocuria polaris*), *Kocuria aegyptia* not only obtained the highest number of gene clusters but also lost the highest number of gene clusters; therefore, it was the most different from the other three bacteria genomes. In the *Kocuria rosea* branch, we obtained a variety of related gene clusters that contribute to transcription (K); defense mechanism (V); carbohydrate transport and metabolism (G); amino acid transport and metabolism (E); and biosynthesis, transport, and catabolism of secondary metabolites (Q). The obtained gene cluster contained aldehyde dehydrogenase (cluster 6298) related to biosynthesis, transport, and catabolism of secondary metabolites; aromatic ring-opening dioxygenase (cluster 6412); alcohol dehydrogenase (cluster 6423); and 2-polypentenyl-6-methoxyphenol hydroxylase and related flavin adenine dinucleotide-dependent oxidoreductase (cluster 6424) of the LigB family, which were likely to be related to aflatoxin degradation. In addition, the newly obtained gene cluster in the *Kocuria salina* clade contains dipriquinone reductase (cluster 4417), which is related to the biosynthesis, transport, and catabolism (Q) of secondary metabolites; the newly obtained gene cluster of the *Kocuria polaris* branch contains 2-polypentenyl-6-methoxyphenol hydroxylase and related flavin adenine dinucleotide-dependent oxidoreductase (cluster 8586). Therefore, three branch strains (*Kocuria rosea*, *Kocuria salina*, and *Kocuria polaris*) of node 10 were found to have new gene clusters related to the metabolism of benzene-containing substances.

### 3.3. Pan-Genome and Core Genome Analysis of the Genus Kocuria Strains

#### 3.3.1. Pan-Genomic Quantitative Analysis

**Figure 3:**
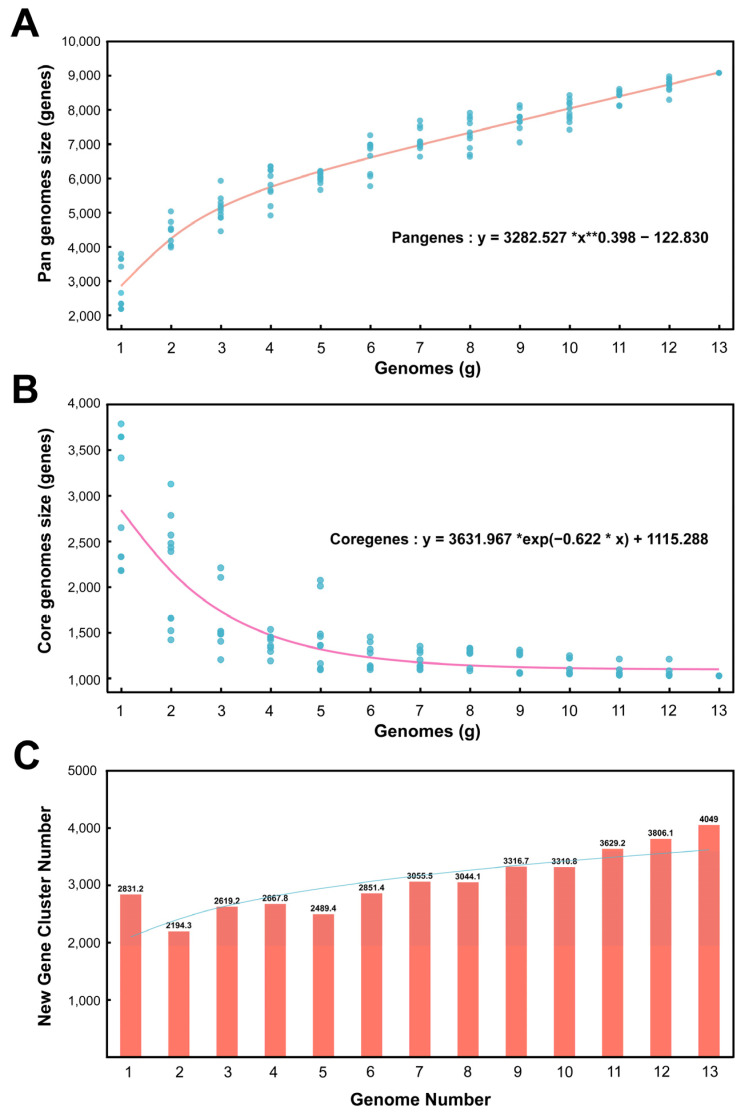
Pan genomes, core genomes, and new gene cluster curves. (**A**) Accumulation curves of the pan genomes. (**B**) Accumulation curves of the core genomes. (**C**) Relationship between new gene cluster and genome number.

According to the software calculation results, there are 9072 homologous gene clusters in the pangenome of 13 *Kocuria* genera and 1024 homologous gene clusters in the core genome. Figure 3A shows the pangenome size changing with the genome number. In the pan-genome calculation formula, the B value is 0.398 greater than 0 and less than 1; therefore, the pan-genome type is open—that is, as the number of genomes increases, the size of the pan-genome would increase indefinitely. Figure 3B shows the core genome size changing with the number of genomes. As the number of genomes increases, the number of core genomes gradually decreases and tends to be flat. Figure 3C is a bar chart of the new gene cluster size changing with the number of genomes. The new gene is the number of gene clusters increased for each additional genome for pan-genomic analysis. It can be seen from Figure 3C that, as the number of genomes increases, the number of new genes gradually increases.

#### 3.3.2. Classification and Distribution of Homologous Genes

**Figure 4:**
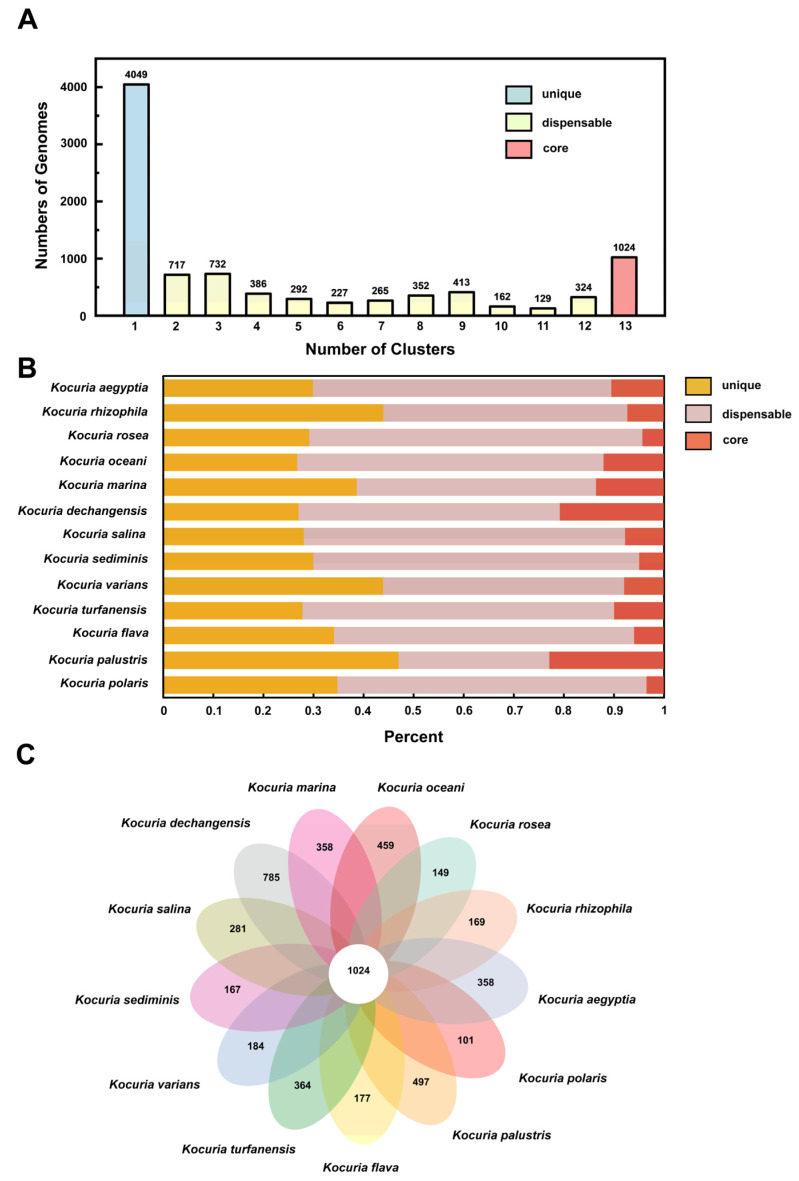
Classification and distribution of homologous genes. (**A**) Numbers of homologous genes in the clusters. (**B**) Distribution of gene clusters in different classifications. (**C**) Pan-genome Venn diagram.

The number of homologous genes in the genome is shown in Figure 4A. There are 4049 unique gene clusters, 3999 non-essential gene clusters, and 1024 core gene clusters in the genome. Figure 4B shows the distribution of three types of gene clusters in the genomes of different strains. Among the 13 strains, 26.8–47.0% of the protein-coding genes were core genes, which was similar to the proportion of core genes in most bacteria (50%) [50]. Figure 4C is a pan-genome Venn diagram, which can visually show the number of core genes, unique genes, and the similarity and overlap of homologous genes in the genomes of different strains. *Kocuria dechangensis* contained the most unique gene clusters (785 clusters), accounting for 20.7% of all gene clusters of the strain, while the aflatoxin-degrading strain *Kocuria rosea* in this study contained only 149 unique gene clusters. The number of unique genome clusters and non-essential gene clusters of strains in the same genus and different species were different, reflecting the diversity of *Kocuria* strains in different habitats. The different number of unique genes may be the result of survival in extreme environments and may contribute to the formation of large pan-genome libraries, as bacterial genomes evolve through gene acquisition, gene loss, and genome rearrangement [51].

### 3.4. Comparative Gene Function Analysis of 13 Strains in the Genus Kocuria

**Figure 5:**
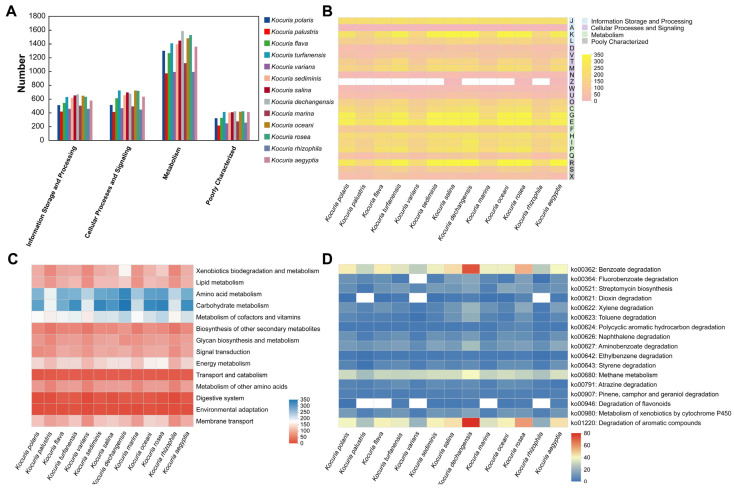
Comparative analysis of gene function in 13 strains of *Kocuria*. (**A**) COG classification comparative heat map. (**B**) COG functional comparison heat map (A: RNA processing and modification; C: energy production and conversion; D: cell cycle control, cell division and chromosome partitioning; E: amino acid transport and metabolism; F: nucleotide transport and metabolism; G: carbohydrate transport and metabolism; H: coenzyme transport and metabolism; I: lipid transport and metabolism; J: translation, ribosomal structure, and biogenesis; K: transcription; L: replication, recombination, and repair; M: cell wall/membrane/envelope biogenesis; N: cell motility; O: posttranslational modification, protein turnover, and chaperones; P: inorganic ion transport and metabolism; Q: secondary metabolites biosynthesis, transport, and catabolism; R: general function prediction only; S: function unknown; T: signal transduction mechanisms; U: intracellular trafficking, secretion, and vesicular transport; V: defense mechanisms; W: extracellular structures; X: mobilome: prophages and transposons; and Z: cytoskeleton). (**C**) KEGG Pathway Level 2 Comparison heat map. (**D**) KEGG Pathway Level 3 Comparison heat map.

The COG functional classifications of the 13 *Kocuria* spp. were compared (Figure 5A). The number of genes in the metabolism was greater in all 13 species than in the other areas (information storage and processing and cellular processes and signaling), and the number of genes for metabolic functions varied greatly among the bacteria within the genus in the metabolism. In addition, in the COG functional comparison heat map (Figure 5B), it was shown that, in terms of information storage and processing, the strains within the genus have small differences in the number of genes involved in translation, ribosomal structure, and biogenesis (J); RNA processing and modification (A); and replication, recombination, and repair (L), while there was a large difference in the transcriptional (K) process. For example, *Kocuria palustris* has only 131 genes involved in the transcription process, whereas *Kocuria dechangensis*, *Kocuria salina*, and *Kocuria oceani* have nearly 300 genes involved in the transcription process, respectively. In addition, in terms of metabolism, this study focuses on the processes of carbohydrate transport and metabolism (G) and secondary metabolites biosynthesis, transport, and catabolism (Q). Among the carbohydrate transport and metabolism (G), *Kocuria rosea* had the most genes involved in this process (297 genes), followed by *Kocuria dechangensis* (293 genes); in the process of secondary metabolites biosynthesis, transport, and catabolism (Q), the *Kocuria rosea* strain also had more genes (87 genes) involved in this process, second only to *Kocuria dechangensis* (97 genes). It indicated that the strains *Kocuria rosea* and *Kocuria dechangensis* are very active in the process of transporting and metabolizing carbohydrates or synthesizing, transporting, and decomposing secondary metabolites.

The Kyoto encyclopedia of genes and genomes (KEGG) functional classification of 13 *Kocuria* genera was compared (Figure 5C), and the functions related to metabolism and transportation were selected from 45 functions, as shown in Figure 5C. The number of genes involved in the processes of heterogeneous biodegradation and metabolism, lipid metabolism, amino acid metabolism, carbohydrate metabolism, cofactor and vitamin metabolism, energy metabolism, and transmembrane transport of different strains of the same genus of *Kocuria* is quite different, indicating that the metabolic processes of the same genus of *Kocuria* strains in the above processes are different to varying degrees. For example, in xenobiotic degradation and metabolism, *Kocuria dechangensis*, *Kocuria rosea*, *Kocuria salina*, and *Kocuria oceani* all have more than 100 genes involved in these processes, whereas strains *Kocuria rhizophila*, *Kocuria palustris*, and *Kocuria varians* have only about 60 genes involved. In carbohydrate metabolism, strains *Kocuria dechangensis*, *Kocuria rosea*, *Kocuria sediminis*, *Kocuria turfanensis*, and *Kocuria aegyptia* had 313–342 genes involved in this metabolism process, while *Kocuria palustris*, *Kocuria varians*, *Kocuria marina*, and *Kocuria rhizophila* had only 189–215 genes involved in this metabolic process. In addition, the number of genes involved in the transport and catabolism, digestive system, and environmental adaptability of the same strain was less different. Compared to most of the other strains of the same genus, the strains *Kocuria rosea* and *Kocuria oceani* were involved in a higher number of genes in the metabolic pathway or transport process, according to Figure 5C.

Further exploration of the pathways and genes related to the metabolism of benzene-containing substances revealed that 13 *Kocuria* strains were involved in 17 pathways related to the metabolism of benzene-containing substances in different degrees in terms of xenobiotic biodegradation and metabolism, energy metabolism, terpenoid and polyketide metabolism, and the biosynthesis of other secondary metabolites (Figure 5D). Compared to other strains in the genus *Kocuria*, strains *Kocuria dechangensis* and *Kocuria rosea* showed a higher number of genes involved in the metabolic pathways of benzoic acid degradation, fluorobenzoate degradation, dioxin degradation, xylene degradation, toluene degradation, polycyclic aromatic hydrocarbons degradation, naphthalene degradation, aminobenzoate degradation, ethylbenzene degradation, styrene degradation, atrazine degradation, and cytochrome P450 for exogenous drugs. In addition, in energy metabolism, 13 strains of *Kocuria* have more than 20 genes involved in methane metabolism. In the metabolic pathway of aromatic compounds, *Kocuria dechangensis* and *Kocuria rosea* had the most genes involved in metabolism, involving 75 and 56 genes, respectively. Therefore, in addition to the AFB_1_-degrading *Kocuria rosea*, the *Kocuria* genus has the ability to metabolize benzene-containing compounds and also has the potential to metabolize AFB_1_.

### 3.5. Analysis of Gene Expression Correlation and Gene Expression Difference

**Figure 6:**
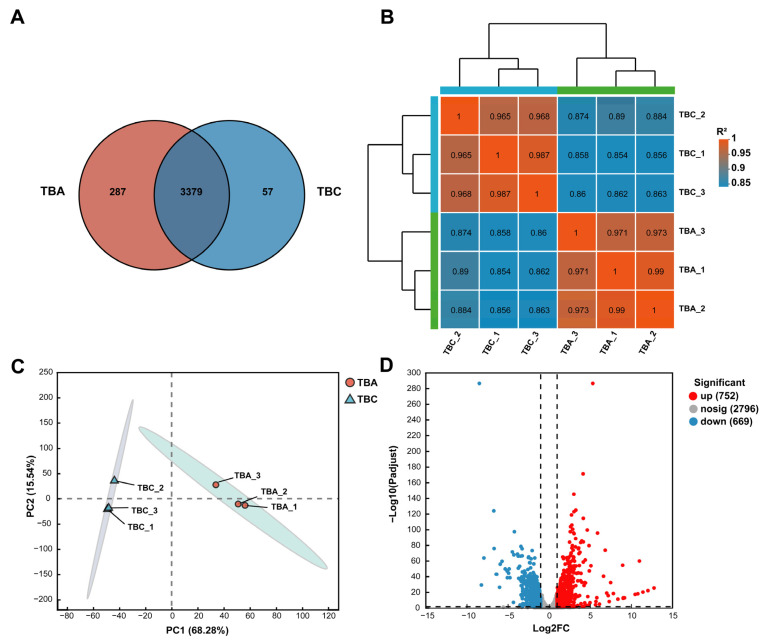
Gene expression analysis. (**A**) Venn analysis. (**B**) Heat map for the correlation analysis between transcriptome samples. (**C**) Principal component analysis. (**D**) Volcano plot of expression differences between the TBA group and TBC group.

Venn analysis can obtain co-expression and specific expression genes between transcriptome groups (TBA vs. TBC). As shown in Figure 6A, there are 3379 genes co-expressed in both the TBA and TBC groups, 287 genes specifically expressed in the TBA group, and only 57 genes specifically expressed in the TBC group. As shown in Figure 6B, the correlation coefficient between TBA-1 and TBA-2 in the TBA group was the highest (0.99), indicating that the expression levels of TBA-1 and TBA-2 in the samples were the closest. In the TBC group, the correlation coefficient between sample TBC-1 and sample TBC-3 was the highest, and the expression results were the most similar. It showed that the correlation between the biological replicates of the same group of samples is high, which verified the rationality of the experimental design. Principal component analysis (PCA) is based on the expression of sample clustering, identifying the impact of sample groups on larger samples. As shown in Figure 6C, principal component (PC) 1 significantly distinguished TBA group and TBC group samples, with a contribution of 68.28%. Additionally, there was only a small difference in PC 2 (contribution 15.54%) between the samples in the TBA group and the TBC group. In addition, according to the number of read counts obtained by gene expression analysis, the differential expression of genes between samples or groups was analyzed, and the differentially expressed genes were identified (Figure 6D). As shown in Figure 6D, a total of 1421 differentially expressed genes (*p*-adjust < 0.05) (Table S4) was found in the samples of the TBA group and TBC group, of which 752 were significantly upregulated and 669 were significantly downregulated. Further study of significantly upregulated genes is of great significance for mining AFB_1_-related degradation genes in *Kocuria rosea*.

### 3.6. Differently Expressed Gene Function Analysis

**Figure 7:**
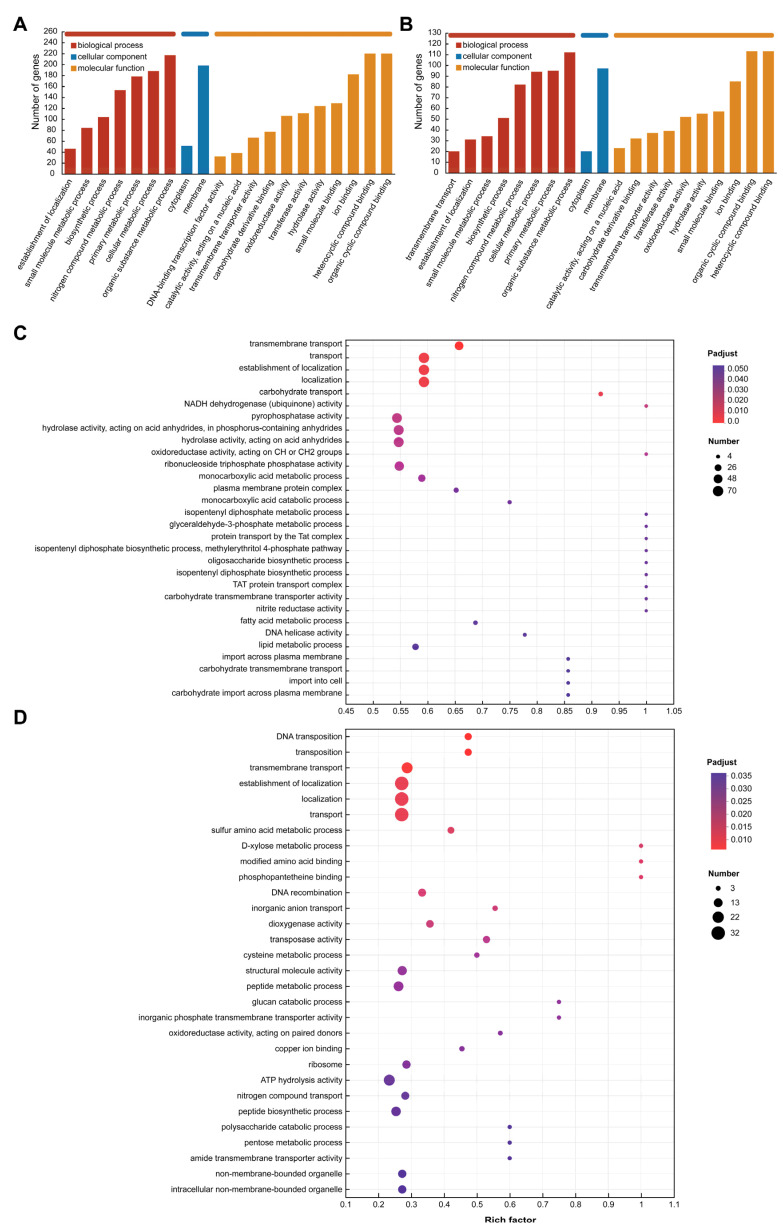
GO functional annotation and functional enrichment analysis. (**A**) GO functional annotation (Level 3) of differentially expressed genes (TBA vs. TBC). (**B**) GO functional annotation (Level 3) of upregulated genes (TBA vs. TBC up). (**C**) GO functional enrichment analysis (Level 4) of differentially expressed genes (TBA vs. TBC). (**D**) GO functional enrichment analysis (Level 4) of upregulated genes (TBA vs. TBC up).

The GO database was used to annotate the functional categories of differentially expressed genes and upregulated genes in the TBA vs. TBC group. The results of Figure 7A,B show that these genes are related to multiple terms in the three clusters, including the biological processes involved, the components of the cells, and the molecular functions. Differentially expressed genes and upregulated genes are mainly involved in molecular functions and biological processes. In terms of biological processes, the differentially expressed genes and upregulated genes involved in the metabolic process of organic matter were the most, with 217 and 112 genes, respectively. In addition, changes in the carbon source of *Kocuria rosea* also caused changes in the expression of related genes in cell metabolic processes, major metabolic processes, nitrogen metabolic processes, biosynthetic processes, and small molecule synthesis. In addition to the above processes, there are 20 genes involved in transmembrane transport. These genes are likely to be related to the way AFB_1_ and its degradation products enter the bacteria. In addition, in terms of molecular function, there were 220 differentially expressed genes involved in organic ring compound binding and heterocyclic compound binding, while 113 upregulated genes were involved in both functions. Nearly 418 differentially expressed genes were involved in the synthesis of hydrolases, transferases, oxidoreductases, and the binding of carbohydrate derivatives, and the upregulated genes were likely to participate in the metabolic process of AFB_1_ in the *Kocuria rosea* strain, with 553,952 and 32 upregulated genes, respectively.

Additionally, Figure 7C,D showed the top 30 GO Term (lever 4) enrichment results of differentially expressed genes and upregulated genes, respectively, under the premise of *p*-adjust < 0.05. In the functional enrichment analysis of the differential genes (Figure 7C), the genes associated with biological processes related to the isopentenyl diphosphate metabolic process (GO ID: 0046490), glyceraldehyde-3-phosphate metabolic process (GO ID: 0019682), protein transport by the Tat complex (GO ID: 0043953), methylerythritol 4-phosphate pathway (GO ID: 0019288), oligosaccharide biosynthetic process (GO ID: 0009312), and isopentenyl diphosphate biosynthetic process (GO ID: 0009240)-related genes were the most highly enriched, and the enrichment factor was 1. In addition, molecular functional classification of NADH dehydrogenase (ubiquinone) activity (GO ID: 0008137), oxidoreductase activity acting on CH or CH_2_ groups (GO ID: 0016725), carbohydrate transmembrane transporter activity (GO ID: 0015144), and nitrite reductase activity (GO ID: 0098809) also had the highest gene enrichment with enrichment factor of 1. In the functional enrichment analysis of the upregulated expressed genes (Figure 7D), biological process with respect to transmembrane transport (GO ID: 0055085), and molecular function with respect to dioxygenase activity (GO ID: 0051213), transferase activity (GO ID: 0004803), oxidoreductase activity (GO ID: 0016705; 0016708), hydrolase activity (GO ID: 0004553), and benzoate 1,2-dioxygenase activity (GO ID: 0018623) involve related genes that may be associated with the transmembrane transport and catabolism of AFB_1_ and its metabolites.

### 3.7. Analysis of AFB_1_ Degradation-Related Genes

The ability of bacteria to tolerate harsh environmental conditions is closely related to the function of their cytosolic pumps, which consist of the following five main categories, including ATP-binding cassette (ABC) superfamily, major facilitator (MFS) superfamily, resistance nodulation-cell division (RND), small multidrug resistance (SMR) superfamily, and multidrug and toxic compound extrusion (MATE) superfamily [52]. Under AFB_1_ stress, multiple ABC superfamily and MFS superfamily genes were identified and significantly upregulated during the culture. It has been reported that ABC transporters and MFS transporters contribute to the transmembrane transport of polybrominated diphenyl ethers [53]. In this study, we found that the expression of seven ABC transporter ATP-binding proteins (such as QR564_00985, QR564_19270 and QR564_02080); six ABC transporter substrate-binding proteins (such as QR564_00990, QR564_15015, and QR564_17590); 13 ABC transporter permeases (such as QR564_00995, QR564_15005, and QR564_15010); and 13 MFS transporters (such as QR564_01195, QR564_02785, and QR564_01110) were significantly upregulated during AFB_1_ biodegradation, which indicated that these proteins are likely to be involved in the transmembrane transport of AFB_1_ and metabolic intermediates. Additionally, ABC transporter proteins and MFS transporter proteins have been involved in the active efflux process, thus strengthening the resistance of microbial cells to various compounds [54]. Therefore, these upregulated transporters may also lead to the tolerance of microbial cells to AFB_1_ and its metabolic intermediates.

Studies have shown that the strain *Kocuria rosea* is mainly involved in the process of hydroxylation, redox, and ring opening in the degradation of AFB_1_ [33]. Cytochrome P450 is a heme-containing metal enzyme with strong oxidizing ability. It has been reported that cytochrome P450 plays a crucial role in the biodegradation of organic compounds, such as polychlorinated biphenyls and polycyclic aromatic hydrocarbons [55,56,57]. In this study, the gene-encoding cytochrome P450 (QR564_01780) was significantly upregulated (4.031-fold increase), indicating that cytochrome P450 may contribute to the hydroxylation of AFB_1_. Therefore, cytochrome P450 may be involved in the degradation of AFB_1_ by *Kocuria rosea*. In addition, gene expressions of oxidase and other metabolic enzymes may be overexpressed during AFB_1_ degradation. For example, the expression of several oxygenase genes, including quinoline monooxygenase (QR564_15695), FAD-dependent monooxygenase (QR564_16670), and LLM class flavin-dependent oxidoreductase (QR564_01200), showed significant upregulation, which may be related to the hydroxylation process of AFB_1_ (Table S5). Moreover, oxygenase, hydrolase, and oxidoreductase may catalyze the ring-opening cleavage of AFB_1_. Under the action of AFB_1_, the ring cleavage dioxygenase (QR564_16850); benzoate 1, 2-dioxygenase (QR564_04565: benA, QR564_04560: benB, and QR564_04555: benC); dienolactone hydrolase (QR564_17010); LLM flavin-dependent oxidoreductase (QR564_03835 and QR564_01200); FAD-binding oxidoreductase (QR564_01985); FAD-dependent oxidoreductase (QR564_01540 and QR564_01865); NAD-dependent oxidoreductase (QR564_01920); and NAD(P)/FAD-dependent oxidoreductase (QR564_01095) were significantly upregulated, indicating dioxygenase, hydrolase, and oxidoreductase may be involved in the biodegradation of AFB_1_.

## 4. Conclusions

The ocean of almost unlimited bacterial species diversity remains an underexplored world. Many bacteria of marine origin play important functions in bioremediation. In this study, we conducted comparative genomic analysis to investigate the evolutionary patterns (evolutionary selection pressure analysis, gene family expansion analysis, pan-genome, and core genome) of the 13 *Kocuria* strains and to explore the biological potential of *Kocuria* bacteria in degrading AFB_1_ in bioremediation. In phylogenetic analysis, *Kocuria rosea*, *Kocuria polaris*, *Kocuria salina*, and *Kocuria aegyptia* are more likely to have similar genes and functions. It was discovered that the *Kocuria* gene family had a purification selection effect, and five clusters involved in positive effects were annotated in DNA-binding transcriptional regulators, the amino acid transport system, carbohydrate transport and metabolism and transcription processes, and inorganic ion transport and metabolism. Moreover, thirteen strains of the genus *Kocuria* are differentially involved in 17 pathways associated with the metabolism of benzene ring-containing substances. Therefore, in addition to the AFB_1_-degrading *Kocuria rosea*, the *Kocuria* genus strains have the ability to metabolize benzene-containing compounds and also have the potential to metabolize AFB_1_. In the transcriptome analysis, a total of 1421 differentially expressed genes were found between the TBA group and TBC group, including 752 significantly upregulated genes, which are of great significance for mining AFB_1_-related degradation genes in *Kocuria rosea*. Nearly 17 upregulated genes were involved in the synthesis of hydrolases, transferases, and oxidoreductases and the binding of carbohydrate derivatives, which may be involved in the metabolism of AFB_1_ by the *Kocuria rosea* strain. The potential biotechnological applications of the strain were explored, which provided a valuable basis for the development of AFB_1_ degradation and bioremediation strategies. However, the degradation gene candidates need to be verified and explored in AFB_1_ degradation muti-steps. Molecular simulation, proteomic technology, metabonomic technology, and gene recombinant techniques could be applied for further AFB_1_ degradation mechanism exploration in the future.

## Figures and Tables

**Table 1:** The 13 strains of the genus *Kocuria* used in this study.

Species	Strain	NCBI Accession No. (IMG ID)	Genome Size (Mb)	GC Content (%)
*Kocuria rosea*	13	CP127857.1	4.2	72.0
*Kocuria varians*	80	CP059343.1	2.8	70.5
*Kocuria rhizophila*	NBC_00781	CP108927.1	2.8	71.0
*Kocuria flava*	HO-9041	CP013254.1	3.6	74.0
*Kocuria palustris*	MU14/1	CP012507.1	2.9	70.5
*Kocuria turfanensis*	HO-9042	CP012507.1	4.2	73.0
*Kocuria oceani*	FXJ8.057	JARAMH000000000.1	4.2	72.5
*Kocuria polaris*	CMS 76or	JSUH00000000.1	3.8	73.0
*Kocuria sediminis*	JCM 17929	WOGU00000000.1	3.9	73.0
*Kocuria marina*	AM104-93	JAQDQP000000000.1	3.1	69.0
*Kocuria aegyptia*	JCM 14735	BAAAOA000000000.1	3.9	72.5
*Kocuria salina*	CV6	SSSF00000000.1	4.2	72.0
*Kocuria dechangensis*	CGMCC 1.12187	BMEQ00000000.1	4.5	72.5

## Data Availability

The transcriptomic sequencing project of *Kocuria rosea* 13 has been deposited in the NCBI under accession number PRJNA1233352, which was used in this study.

## Supplementary Materials

The following supporting information can be downloaded at https://www.mdpi.com/article/10.3390/microorganisms13040875/s1, Table S1: M2 media composition. Table S2: Sequencing data quality control. Table S3: Ka to Ks ratio of homologous genes. Table S4: Gene expression differences. Table S5: Upregulated genes related to the AFB_1_ metabolic process of *Kocuria rosea*. Figure S1: Schematic diagram of the process of preparing the bacterial cells.
