# Supplementary figures and images for: Comparative Genomic and Transcriptomic Analysis Provides New Insights into the Aflatoxin B1 Biodegradability by Kocuria rosea from Deep Sea
Source: Microorganisms. 2025 Apr 10;13(4):875. doi: 10.3390/microorganisms13040875 (PMC12029209; doi:10.3390/microorganisms13040875)

## Seed solution preparation

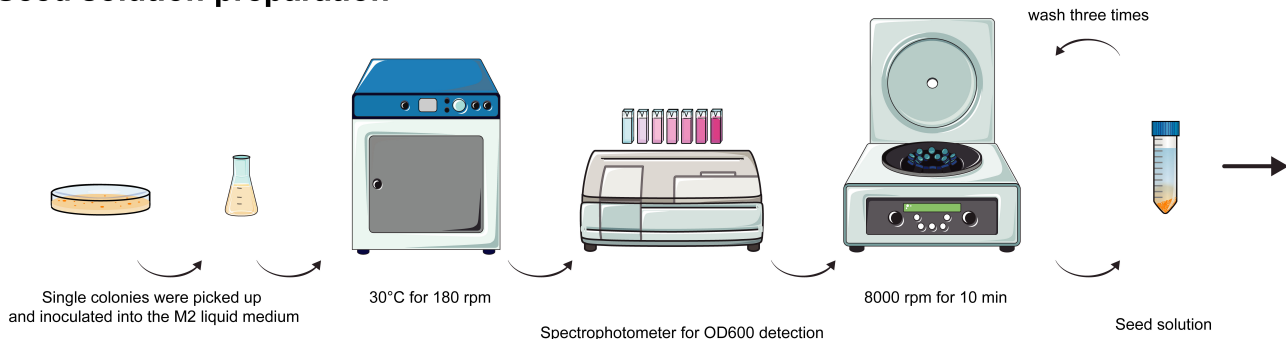

## Induction culture

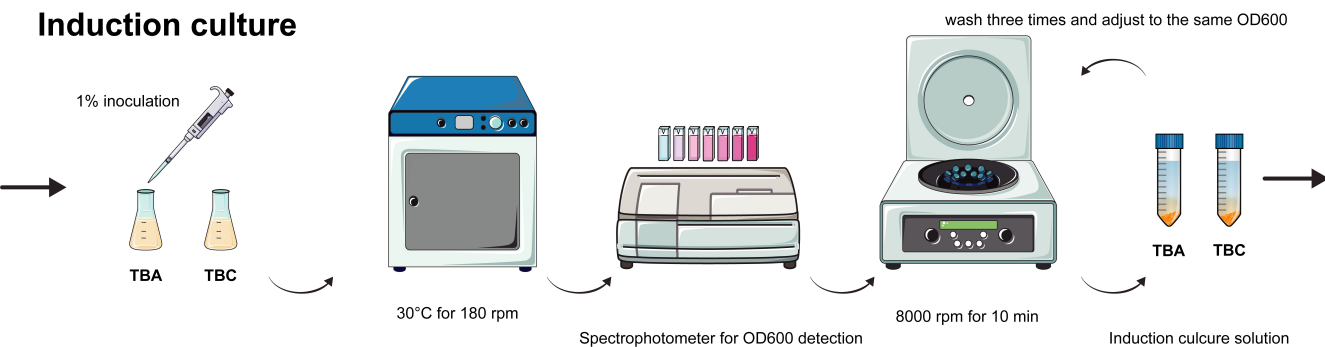

## Large-volume culture

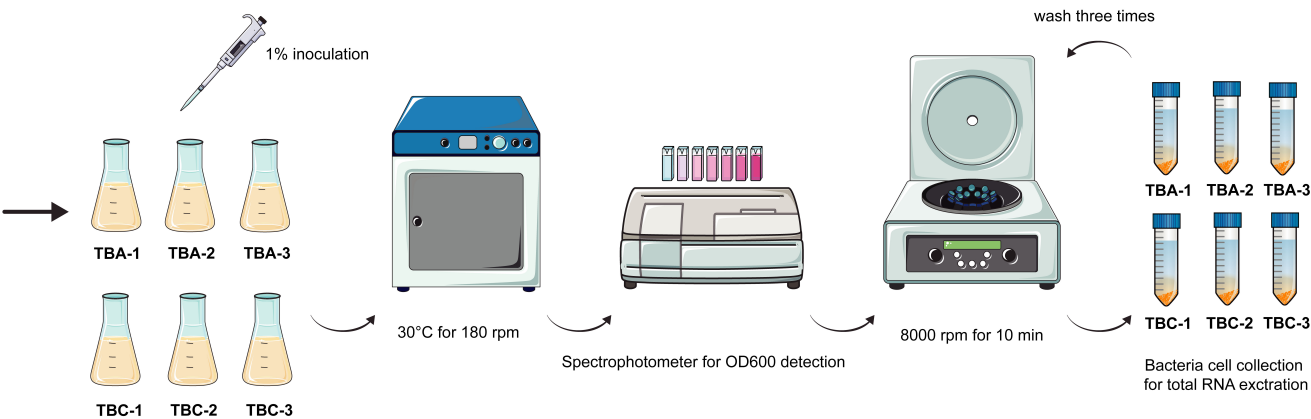

Supplement: Supplementary file 1 [file microorganisms-13-00875-s001.zip › Figure S1.pdf]
